# Monitoring progress towards the first UNAIDS 90-90-90 target in key populations living with HIV in Norway

**DOI:** 10.1186/s12879-020-05178-1

**Published:** 2020-06-26

**Authors:** Robert Whittaker, Kelsey K. Case, Øivind Nilsen, Hans Blystad, Susan Cowan, Hilde Kløvstad, Ard van Sighem

**Affiliations:** 1 Norwegian Institute of Public Health, Lovisenberggata 8, 0456 Oslo, Norway; 2 European Program for Intervention Epidemiology Training, European Centre for Disease Prevention and Control, Stockholm, Sweden; 3 Department of Infectious Disease Epidemiology, Imperial College London, London, UK; 4 Statens Serum Institut, Copenhagen, Denmark; 5 Stichting HIV Monitoring, Amsterdam, The Netherlands

**Keywords:** HIV, AIDS, Incidence, Prevalence, Norway, Statistical models

## Abstract

**Background:**

In line with the Joint United Nations Programme on HIV/AIDS (UNAIDS) 90-90-90 target, Norway aims for at least 90% of people living with HIV (PLHIV) to know their HIV-status. We produced current estimates of the number of PLHIV and undiagnosed population in Norway, overall and for six key subpopulations: Norwegian-born men who have sex with men (MSM), migrant MSM, Norwegian-born heterosexuals, migrant Sub-Saharan Africa (SSA)-born heterosexuals, migrant non-SSA-born heterosexuals and people who inject drugs.

**Methods:**

We used the European Centre for Disease Prevention and Control (ECDC) HIV Modelling Tool on Norwegian HIV surveillance data through 2018 to estimate incidence, time from infection to diagnosis, PLHIV, and the number and proportion undiagnosed. As surveillance data on CD4 count at diagnosis were not collected in Norway, we ran two models; using default model CD4 assumptions, or a proxy for CD4 distribution based on Danish national surveillance data. We also generated alternative overall PLHIV estimates using the Spectrum AIDS Impact Model, to compare with those obtained from the ECDC tool.

**Results:**

Estimates of the overall number of PLHIV in 2018 using different modelling approaches aligned at approximately 5000. In both ECDC models, the overall number undiagnosed decreased continuously from 2008. The proportion undiagnosed in 2018 was lower using default model CD4 assumptions (7.1% [95%CI: 5.3–8.9%]), than the Danish CD4 proxy (10.2% [8.3–12.1%]). This difference was driven by results for heterosexual migrants. Estimates for Norwegian-born MSM, migrant MSM and Norwegian-born heterosexuals were similar in both models. In these three subpopulations, incidence in 2018 was < 30 new infections, and the number undiagnosed had decreased in recent years. Norwegian-born MSM had the lowest estimated number of undiagnosed infections (45 [30–75], using default CD4 assumptions) and undiagnosed fraction (3.6% [2.4–5.7%], using default CD4 assumptions) in 2018.

**Conclusions:**

Results allow cautious confidence in concluding that Norway has achieved the first UNAIDS 90–90-90 target, and clearly highlight the success of prevention strategies among MSM. Estimates for subpopulations strongly influenced by migration remain less clear, and future modelling should appropriately account for all-cause mortality and out-migration, and adjust for time of in-migration.

## Background

While infection with HIV continues to be a major global public health issue [1], antiretroviral treatment (ART) has successfully reduced morbidity and mortality in people living with HIV (PLHIV) [2–4], and prevented onward transmission [5–7]. This provides the basis for the Joint United Nations Programme on HIV/AIDS (UNAIDS) 90–90-90 target for ending the AIDS epidemic by 2030, the first of which aims for 90% of PLHIV to know their HIV status by 2020 [8]. In addition to reliable data on the number of HIV diagnoses, a robust estimate of the number of undiagnosed HIV infections is required to monitor the first target. This can provide information on current gaps in testing programmes, particularly among key subpopulations, which can inform strategies to reach those undiagnosed [8].

Robust case surveillance and vital registration data supports back-calculation approaches to estimate the number of PLHIV and undiagnosed population. Several such approaches have been developed in recent years [9–15]. The majority of countries in the European region of the World Health Organisation have produced national estimates using either the HIV modelling tool from the European Centre for Disease Prevention and Control (ECDC) [16], or the AIDS Impact Model in Spectrum (denoted herein as ‘Spectrum’) [12]. Both tools are freely available and recommended in Europe. The ECDC modelling tool can model key subpopulations, in addition to the general epidemic [16].

Norway is a low HIV prevalence country with a longstanding surveillance system. Clinical AIDS diagnoses have been under mandatory nominal reporting to the Norwegian Institute of Public Health (NIPH) since 1983. Diagnoses of HIV were subject to mandatory, anonymised reporting by clinicians and laboratories since 1986, with diagnoses from 1984 and 1985 reported retrospectively. Reporting on HIV diagnoses was made nominal, and the collection of data on CD4 cell count at diagnosis included, from March 2019 [17]. In Norway, residents diagnosed with HIV can access ART free of charge [18]. Pre-exposure prophylaxis (PrEP) has been available since January 2017 [17]. Key risk groups to which specific testing offers are directed include men who have sex with men (MSM), migrants from HIV endemic areas and people who inject drugs (PWID) [19, 20].

There were 6468 HIV diagnoses reported to the NIPH from 1984 to 2018 [17]. Initially, the epidemic was characterised by diagnoses amongst MSM and PWID. Diagnoses peaked in 2008 (*n* = 299), driven by increased migration from high-endemic areas like Sub-Saharan Africa (SSA) in the early 2000s [21–23] and an increase in diagnoses among MSM. Diagnoses have declined recently (*n* = 191 in 2018), driven by decreases among both Norwegian-born (those born in Norway, and/or of Norwegian origin) MSM, and heterosexual migrants who acquired HIV before migration to Norway. Conversely, there has been an increasing number of diagnoses among migrant MSM reported to have acquired infection before migration. A low, stable number of diagnoses continues to be reported among PWID, heterosexual Norwegian-born, and heterosexual and migrant MSM resident in Norway at the time of infection [17].

There have been few measures of the HIV epidemic in Norway. A back-calculation approach was used to estimate PLHIV and the undiagnosed population up to the year 2000 [22, 24]. More recent estimates have been generated by UNAIDS, using the ECDC modelling tool to calculate national incidence, and Spectrum to calculate prevalence. The most recent estimate, using surveillance data through 2017, was 5800 PLHIV (uncertainty bounds 5200–6300), however, the input data for these estimates incorrectly included cases previously diagnosed in another country, before re-diagnosis upon arrival in Norway [15, 25].

We aimed to produce current estimates of the number of PLHIV and the undiagnosed population in Norway, overall and for six key subpopulations, to monitor progress towards the first UNAIDS 90–90-90 target. The six key subpopulations were; Norwegian-born MSM, migrant (those not born in Norway, and not of Norwegian origin) MSM, Norwegian-born heterosexuals, migrant SSA-born heterosexuals, migrant non-SSA-born heterosexuals and PWID.

## Methods

### Data preparation and cleaning

We extracted records of all notified HIV and clinical AIDS diagnoses through 2018 from the Norwegian Surveillance System for Communicable Diseases. We categorised an HIV diagnosis for which the reported clinical picture was AIDS as a concurrent HIV/AIDS diagnosis. We excluded diagnoses among persons aged < 15 years, diagnoses of HIV-2 and diagnoses among persons known to have previously had a positive HIV diagnosis in another country. Records were stratified by reported route of transmission and region of birth (see Additional file 1, Table A).

### ECDC modelling tool

The incidence method in the ECDC HIV Modelling Tool version 1.3.0 is a multi-state back-calculation model based on maximum likelihood statistics [11, 16]. The method uses routinely collected HIV surveillance data to estimate new HIV infections over time, using cubic B-splines for parametrising the incidence curve, and time from infection to diagnosis by CD4+ cell count strata (≥ 500, 350–499, 200–349, < 200), incorporating user-defined intervals to specify the probability of diagnosis over time. The model then estimates HIV prevalence and the undiagnosed population. In-migration is not taken into account. The model captures pre-1995 AIDS-related mortality in people with AIDS. Observed data on out-migration and all-cause mortality are used to estimate the number still living in the country.

The input data used in the ECDC modelling tool are presented in Table 1. The model was based on HIV and HIV/AIDS diagnoses from 1987, following the early peak in diagnoses related to the introduction of HIV testing. We included AIDS diagnoses from 1983, the first year with mandatory reporting. Data on CD4 count at diagnosis were not reported to the NIPH in the study period. Given the similarities in HIV epidemics, target groups for testing, accessibility of testing, and the availability and accessibility of treatment [19, 20, 27–32], we used Danish national surveillance data on CD4 count at diagnosis as a proxy for the Norwegian CD4 distribution, adjusting for transmission route (including gender for heterosexual transmission) and region of birth. We used Danish CD4 count data from 2004 onwards, the first year with sufficient completeness for it to be representative (Table 1). We ran two models, using either default model assumptions for CD4 distribution in the absence of CD4 data for the whole study period, or default model assumptions until 2003 and the Danish CD4 proxy from 2004 to 2018. We adjusted the number of knots to obtain improvements in the spline fit. We generated 95% confidence intervals (CI) through bootstrap analysis (100 iterations). The parameters used in the tool and resulting model fits are presented in Additional file 2.

**Table 1:** Input data used for running the ECDC HIV modelling tool and CSAVR tool in Norway

Model	Input data item	Data source
ECDC HIV modelling tool	New HIV diagnoses^a^	National surveillance data from 1987 to 2018 (*n* = 5318)
	New AIDS diagnoses	National surveillance data from 1983 to 1995^b^ (*n* = 481)
	New HIV/AIDS^c^ diagnoses	National surveillance data from 1987 to 2018 (*n* = 1140)
	CD4 count at diagnosis among non-HIV/AIDS diagnoses categorised into four strata (≥500, 350–499, 200–349, < 200)	Default model assumptions on CD4 distribution for whole study period OR Default model assumptions until 2003 and Danish national CD4 count data from 2004 to 2018, adjusted for route of transmission and region of birth^d^.
	All-cause mortality and outmigration	National surveillance data from 1987 to 2018 (*n* = 759)^e^
CSAVR tool	New HIV diagnoses^a^	National surveillance data from 1987 to 2018 (*n* = 5318)
	AIDS deaths	Adjusted estimates of AIDS-related deaths from 1990 to 2017 (*n* = 703)^f^

^a^ All new HIV diagnoses, including those with AIDS at first diagnosis ^b^ AIDS diagnoses after 1995 were not used because the probability of progressing to AIDS would be affected by the use of combination ART, the effect of which is not incorporated into the model. ^c^ HIV diagnosis for which the reported clinical picture was AIDS. ^d^ Before 2004, the data completeness for CD4 count in Denmark was < 10%. In 2004 it was 48%, and over 80% from 2009 onwards (88% in 2018). ^e^ Data on outcome was known for 14% of HIV diagnoses (*n* = 759), all of which were reported as dead or out-migrated. ^f^ Estimates produced by the Institute of Health Metrics and Evaluation for the Global Burden of Disease Study [26]

After running the national model, we used the same settings to separately model the six key subpopulations. We did not further disaggregate subpopulations by region of birth, sex or other routes of transmission due to the low number of diagnoses (see Additional file 1, Table A).

### Spectrum

For comparison of national estimates, we used the Spectrum AIDS Impact Model version 5.76 to calculate two PLHIV estimates, one using the HIV incidence estimate from the Case Surveillance and Vital Registration (CSAVR) tool, and one importing incidence from the ECDC modelling tool to take advantage of the mortality assumptions in Spectrum instead of relying on reported outcomes.

Estimation of incidence using the CSAVR tool in Spectrum has been previously described [12, 33, 34]. Briefly, routine reporting of new diagnoses, mean CD4 at diagnosis and AIDS deaths are used for model fitting. Four curve fitting options are available that provide flexibility to capture different epidemic trajectories. The best fitting curve is informed by Akaike information criteria (see Additional file 2). We fit to new diagnoses from 1987 to 2018, and estimates of AIDS deaths adjusted for misclassification and completeness (Table 1). To calculate PLHIV in Spectrum, estimates of incidence are disaggregatedby age, sex and CD4 count then the newly infected population is tracked over time, using patterns of CD4 progression and mortality by treatment status [35, 36].

The underlying demographic projections in Spectrum are produced by the United Nations Population Division (World Population Prospects 2017 Revision) and include patterns of fertility, mortality and migration. The effect of net migration on the number of PLHIV is incorporated using the assumption migrants have the same HIV prevalence as the resident population. Program statistics were provided by national public health officials for numbers on ART over time. We used all Spectrum default model parameters for developed countries but updated assumptions for the sex ratio of incidence over time based on surveillance data in Norway.

## Results

### Overall

**Fig. 1:**
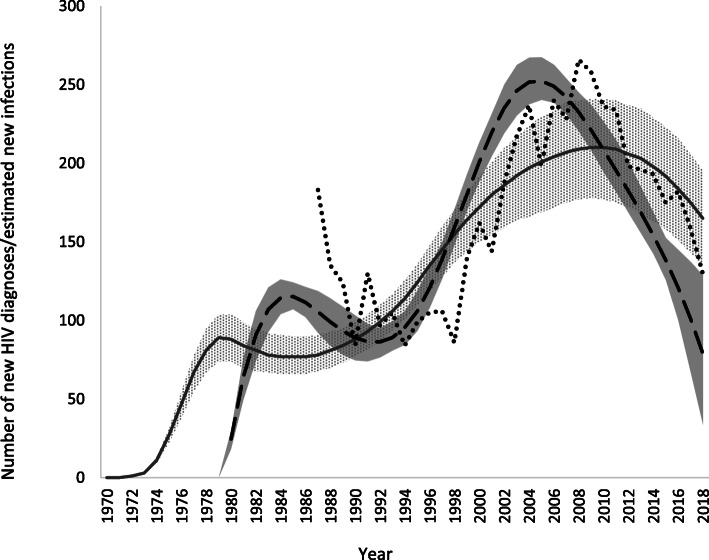
Number of new HIV diagnoses, and estimated HIV incidence curves, by year, Norway. Dotted line: Input data on new HIV diagnoses used in the models. Dashed line: Estimates of incidence from the ECDC HIV modelling tool using default model assumptions for CD4 distribution. Solid area: 95% confidence interval from the ECDC HIV modelling tool using default model assumptions for CD4 distribution. Solid line: Estimates of incidence from the CSAVR tool. Dotted area: Uncertainty bound from the CSAVR tool

**Fig. 2:**
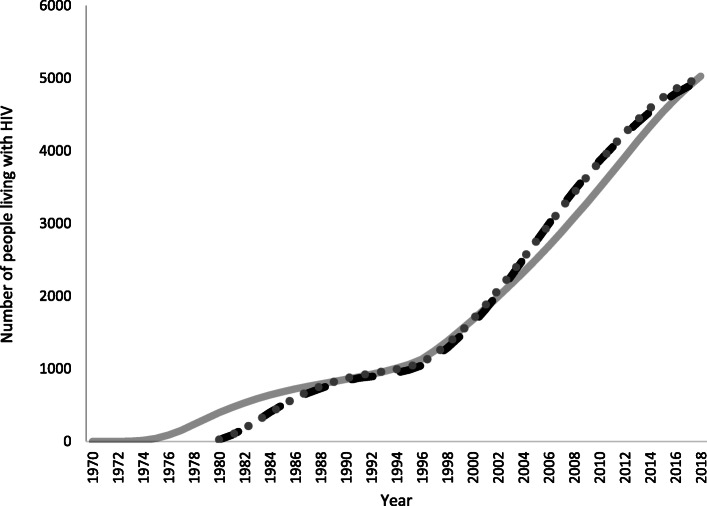
Estimated number of people living with HIV by year using different estimation methods, Norway. Dashed line: ECDC HIV modelling tool using default model assumptions for CD4 distribution. Solid line: Spectrum using the CSAVR tool. Dotted line: Spectrum using incidence estimates from the ECDC HIV modelling tool using default model assumptions for CD4 distribution. Estimates exclude diagnoses among persons previously diagnosed in another country who were not reported to have died or out-migrated by the end of 2018. The Spectrum models incorporate previous positive cases in the PLHIV estimate through the assumption that migrants have the same HIV prevalence as the resident population

**Fig. 3:**
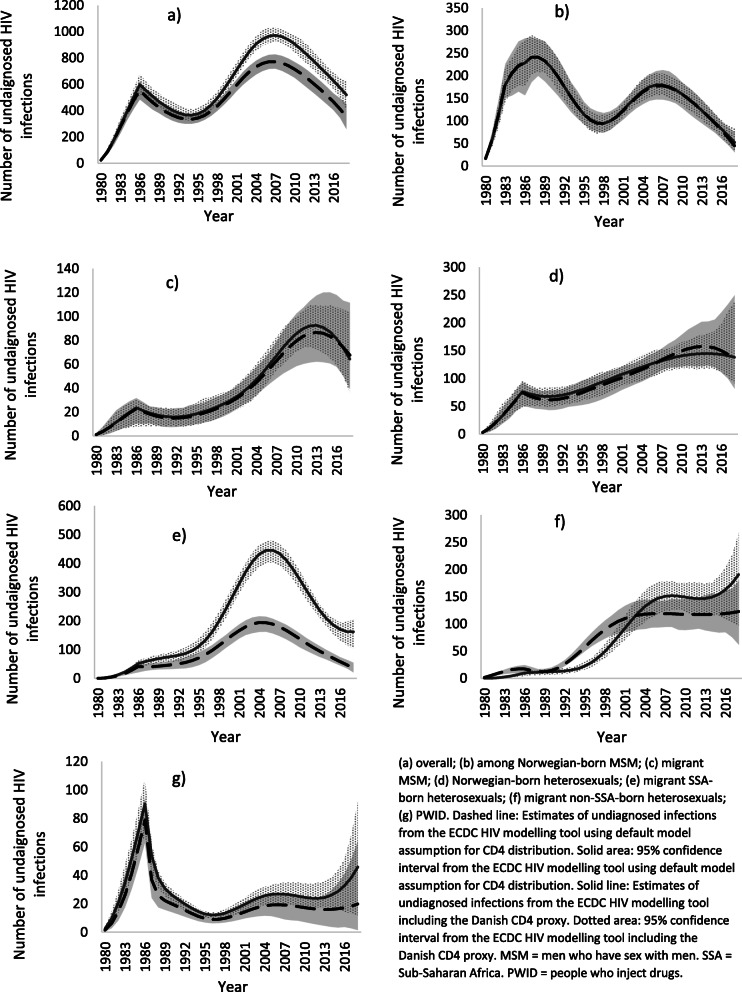
Estimated undiagnosed HIV infections overall and by key subpopulation, Norway, 1980–2018. **a** overall; **b** among Norwegian-born MSM; **c** migrant MSM; **d** Norwegian-born heterosexuals; **e** migrant SSA-born heterosexuals; **f** migrant non-SSA-born heterosexuals; **g** PWID. Dashed line: Estimates of undiagnosed infections from the ECDC HIV modelling tool using default model assumption for CD4 distribution. Solid area: 95% confidence interval from the ECDC HIV modelling tool using default model assumption for CD4 distribution. Solid line: Estimates of undiagnosed infections from the ECDC HIV modelling tool including the Danish CD4 proxy. Dotted area: 95% confidence interval from the ECDC HIV modelling tool including the Danish CD4 proxy. MSM = men who have sex with men. SSA = Sub-Saharan Africa. PWID = people who inject drugs

The ECDC model using default model assumptions for CD4 distribution estimated 79 new HIV infections in Norway in 2018 (95%CI: 34–129) (Table 2, Fig. 1). The number of new infections decreased continuously from a peak in 2005 (252 infections [95%CI: 240–268]). Since 2008, there were fewer estimated infections than diagnoses per year (Fig. 1). The median time to diagnosis was 2.2 years (interquartile range: 1.1–4.1) in 2018 (Table 2). There were an estimated 4964 (95%CI: 4789–5181) PLHIV in 2018 (Table 2, Fig. 2)). The number of undiagnosed infections had decreased consistently from a peak of 773 (719–827) in 2007 (Fig. 3). There were an estimated 355 undiagnosed infections (259–449) in 2018 (Table 2, Fig. 3) which yields an undiagnosed fraction of 7.1% (5.3–8.9) (Table 2). Using the Danish CD4 proxy had a minimal effect on the estimates of the number of new infections and PLHIV, but resulted in a longer estimated time from infection to diagnosis (3.0 years, interquartile range: 1.5–5.7), a higher number of undiagnosed infections (520 [95%CI: 411–627]), and thus a larger undiagnosed fraction (10.2% [8.3–12.1]) (Table 2).

**Table 2:** ECDC HIV modelling tool results, excluding and including CD4 count proxy from Denmark, Norway, 2018

Population	Number of new HIV infections (95%CI)	Median number of years from infection to diagnosis (interquartile range)	Number of PLHIV^a^ (95%CI)	Number of undiagnosed HIV infections (95%CI)	Proportion undiagnosed (95%CI)
**Using default model assumptions for CD4 distribution**					
Overall	79 (34–129)	2.2 (1.1–4.1)	4964 (4789–5181)	355 (259–449)	7.1% (5.3–8.9)
Norwegian-born MSM	9 (5–25)	1.7 (0.8–3.1)	1245 (1163–1350)	45 (30–75)	3.6% (2.4–5.7)
Migrant MSM	21 (4–36)	2.0 (1.0–3.7)	495 (446–568)	68 (39–111)	13.7% (8.6–20.1)
Norwegian-born heterosexuals	19 (4–55)	3.9 (1.9–7.0)	864 (794–1009)	134 (81–250)	15.5% (10.2–24.8)
Migrant SSA-born heterosexuals	13 (5–27)	1.3 (0.7–2.4)	1544 (1451–1641)	35 (21–55)	2.3% (1.3–3.6)
Migrant non-SSA-born heterosexuals	31 (4–52)	3.3 (1.6–6.0)	649 (558–737)	123 (62–180)	18.9% (10.7–25.3)
PWID	6 (1–22)	3.1 (1.5–5.6)	244 (185–308)	20 (2–64)	8.2% (0.8–21.7)
**Using Danish CD4 proxy**					
Overall	86 (29–143)	3.0 (1.5–5.7)	5080 (4898–5262)	520 (411–627)	10.2% (8.3–12.1)
Norwegian-born MSM	13 (5–28)	1.7 (0.9–3.2)	1239 (1151–1351)	52 (33–82)	4.2% (2.6–6.2)
Migrant MSM	16 (4–35)	2.0 (1.0–3.9)	494 (445–558)	64 (36–103)	13.0% (8.4–19.0)
Norwegian-born heterosexuals	28 (5–62)	3.5 (1.7–6.3)	866 (799–1001)	138 (91–238)	16.0% (10.7–24.0)
Migrant SSA-born heterosexuals	28 (10–44)	4.3 (1.9–8.1)	1673 (1592–1788)	162 (105–206)	9.7% (6.4–12.3)
Migrant non-SSA-born heterosexuals	41 (6–78)	5.4 (2.7–9.6)	711 (598–812)	191 (95–268)	26.8% (15.9–34.5)
PWID	12 (1–32)	5.7 (2.7–10.0)	257 (206–328)	46 (12–92)	17.9% (5.9–27.3)

Overall: 5318 HIV diagnoses in the input data, 41% reported to be infected before migration to Norway; Norwegian-born MSM: 1332; migrant MSM: 465, 33%; Norwegian-born heterosexuals: 814; migrant SSA-born heterosexuals: 1625, 94%; migrant non-SSA-born heterosexuals: 548, 78%; PWID: 414, 12%. We did not model all routes of transmission due to the low number of cases for which another known route of transmission was reported. Thus, the sum of the number of HIV diagnoses in the input data for the key subpopulations does not add up to the overall number of HIV diagnoses in the input data. See Additional file 1, Table A for a breakdown of the input data by region of birth, sex and route of transmission. Also, as estimates are done separately for each key subpopulation, the sum of the number of new infections or undiagnosed infections for the key subpopulations may differ from the corresponding estimates for the overall population. ^a^ Excludes diagnoses among persons previously diagnosed in another country who were not reported to have died or out-migrated by the end of 2018. *MSM* men who have sex with men, *SSA* Sub-Saharan Africa, *PWID* people who inject drugs, *PLHIV* people living with HIV

In Spectrum, the best fitting models in the CSAVR tool were produced using the spline and double logistic functions. The spline qualitatively produced the best fit to the recent data. There were an estimated 165 (uncertainty bounds: 135–195) new infections in 2018, decreasing from a peak of 210 (177–241) in 2010. Following the peak, estimates of new infections were slightly higher than diagnoses per year (Fig. 1). In this model, there were an estimated 5030 (4360–5702) PLHIV in 2018 (Fig. 2). When importing the estimated incidence curve from the ECDC modelling tool, the estimate of PLHIV in 2018 was 5002 (4506–5555) (Fig. 2).

### Key subpopulations

The ECDC modelling tool was used to produce estimates among key subpopulations. For Norwegian-born MSM, migrant MSM and Norwegian-born heterosexuals, estimates were similar in the model using default assumptions for CD4 distribution, or the Danish CD4 proxy. Results described are for the model using default assumptions. Of the three subpopulations, Norwegian-born MSM had the lowest estimate of new HIV infections (9 [95%CI: 5–25]), time from infection to diagnosis (1.7 years [interquartile range: 0.8–3.1]) and number of undiagnosed infections (45 [95%CI: 30–75]) in 2018 (Table 2). Estimates were fractionally higher for migrant MSM. Norwegian-born heterosexuals had the longest median time to diagnosis out of the three subpopulations (3.9 years [interquartile range: 1.9–7.0]), and highest number of undiagnosed infections (134 [95%CI: 81–250]) (Table 2, Fig. 3). In all three subpopulations the number of undiagnosed infections decreased consistently, from 2008 for Norwegian-born MSM, and from 2014 for migrant MSM and Norwegian-born heterosexuals (Fig. 3). Norwegian-born MSM had a lower proportion undiagnosed (3.6% [2.4–5.7]) than both migrant MSM (13.7% [8.6–20.1]) and Norwegian-born heterosexuals (15.5% [10.2–24.8]).

Among both migrant SSA-born and non-SSA-born heterosexuals, among whom the majority of new diagnoses were persons reportedly infected before migration to Norway, using the Danish CD4 proxy resulted in a longer median time from infection to diagnosis, and a larger number of undiagnosed infections and proportion undiagnosed. This effect was most pronounced among migrant SSA-born heterosexuals, in which point estimates in the model using the Danish CD4 proxy were outside the 95%CIs of the model using default assumptions for CD4 distribution, and vice versa. Results for PWID were also affected by the use of default CD4 assumptions or the Danish CD4 proxy, although in both models the 95%CIs were wide and estimates of new infections and number undiagnosed in 2018 low (Table 2, Fig. 3).

Detailed results for all models are available in Additional files 3, 4, 5 and 6.

## Discussion

Estimates of the number of PLHIV in Norway by the end of 2018 using the ECDC modelling tool, and Spectrum (with incidence from both CSAVR and the ECDC modelling tool) were all aligned at around 5000 persons. The estimates in this study were generated excluding cases previously diagnosed in another country and re-diagnosed upon migration to Norway and are thus lower than those previously produced for Norway [25]. General agreement in the PLHIV estimates produced using these two models was also observed in a study from French Guiana [37].

From 2008, the overall number of undiagnosed infections decreased. This reflects a peak in new infections and steadily decreasing time from infection to diagnosis among MSM (see Additional files 5 and 6). Analyses from the Netherlands [38] and Switzerland [39] suggest that an increase in infections among MSM up to the mid-2000s was the result of increased high risk sexual behaviour following the introduction of ART, as well as improvements in testing leading to earlier diagnosis, which, alongside better treatment options becoming available [40], led to an eventual reduction in new infections. This decreasing number of undiagnosed infections also reflects changing migration patterns, with a decreasing number of arrivals from high endemic countries in the latter part of the first decade of the twenty-first century [21, 23].

Overall point estimates of the proportion undiagnosed in 2018 using the ECDC modelling tool ranged from 7.1% (355 undiagnosed infections) using default model assumptions for CD4 distribution, to 10.2% (520 undiagnosed infections) with the Danish CD4 proxy, which is amongst the lowest in Europe, and comparable to estimates from neighbouring Denmark and Sweden [13, 15, 41, 42]. National surveillance data and clinical data from the capital, Oslo, support that there is a low proportion undiagnosed in Norway, with a decreasing total number of first-time diagnoses, low proportion of late diagnoses and high proportion achieving viral suppression [17, 27]. Among Norwegian-born heterosexuals and both MSM subpopulations, estimates did not change noticeably with the use of the Danish CD4 proxy. This may provide greater confidence in estimates for these subpopulations, for which there were < 30 estimated new infection in 2018, undiagnosed infections had decreased in recent years, and who collectively had an undiagnosed fraction of 9.5% (247 undiagnosed infections) using default model assumptions, and 9.7% (254 undiagnosed infections) with the Danish CD4 proxy. Among PWID, the Danish CD4 proxy resulted in slightly higher estimates, although interval estimates were wide. Strategies to prevent HIV transmission in PWID are long-standing in Norway [43] and estimates indicate the incidence and number of undiagnosed are low. These results collectively allow cautious confidence in concluding that Norway has achieved the first UNAIDS 90-90-90 target.

Among Norwegian-born MSM, the undiagnosed fraction in 2018 was around 4% with approximately 50 undiagnosed infections. Migrant MSM had an undiagnosed fraction of around 13%, with a fractionally higher estimated incidence and number of undiagnosed infections than Norwegian-born MSM, but estimates were low. This highlights the success of the prevention strategy among MSM in recent years in Norway, which has been to increase awareness of HIV infection and condom use, increase HIV testing in higher risk settings or partnerships, rapid initiation of treatment and, more recently, the implementation of PrEP for at-risk persons [17, 20]. Other European countries have reported higher undiagnosed rates of HIV among migrant MSM [13, 41], and the proportion of MSM infected overseas, and/or with migrant background is increasing in Norway, as well as neighbouring Denmark and Sweden [17, 44, 45]. This highlights the importance of continuing to consider migrants of all backgrounds in prevention efforts, particularly in a country which saw a notable influx of migrants from HIV endemic areas in the first decade of the twentieth century. This includes ensuring the accessibility and availability of testing, early treatment and PrEP. Migrants arriving in Norway from HIV endemic areas are offered an HIV test within 3 months of arrival, and as residents of Norway have free access to ART.

Among Norwegian-born heterosexuals, there was a higher number of undiagnosed infections, time from infection to diagnosis and undiagnosed fraction compared to the two MSM subpopulations. National surveillance data suggest that a higher proportion of MSM diagnosed with HIV have been tested on their own initiative or as part of a routine health check, whilst Norwegian-born heterosexuals diagnosed with HIV are more often tested due to the onset of clinical symptoms and signs of HIV infection [17]. Increased awareness of HIV infection, condom use, and early diagnosis and treatment are the most important preventive measures among heterosexuals. In addition, health care providers should consider informing heterosexual clients who disclose high risk behaviour about PrEP.

Estimates for migrant SSA-born and non-SSA-born heterosexuals were characterised by a high proportion of diagnoses among persons infected before migration to Norway, and results varied depending on the use of the Danish CD4 proxy. Estimates of the number of new infections in these subpopulations do not necessarily reflect incidence in Norway, but rather an extrapolation that depends on patterns of migration. Spectrum accounts for the effect of net migration through the assumption that migrants have the same HIV prevalence as the resident population. For Norway, this may not be the case, as there has been considerable migration from countries with much higher HIV prevalence since the early 2000s [21–23]. For the ECDC modelling tool, in-migration is not taken into account. Also, using CD4 count data in modelling subpopulations with a high proportion infected before migration may overestimate the number undiagnosed, as the time from infection to diagnosis will also include the time before the undiagnosed individual migrated, when they could not conceivably be diagnosed by the receiving country. On the other hand, when CD4 count data are not used (using default CD4 assumptions), estimates of the time from infection to diagnosis are not very robust, as they rely only on data of HIV/AIDS and AIDS diagnoses (ECDC modelling tool) or AIDS deaths (CSAVR).

Ideally, data for both CD4 count at diagnosis, and either precise migration data or an appropriate assumption on migration, should be incorporated to more appropriately generate estimates in populations with high levels of in-migration [44]. In Norway, the history of anonymised reporting reduces the potential to use precise migration data, while surveillance data on CD4 count were not collected prior to March 2019. However, the recent introduction of nominal reporting, collection of CD4 data, and a planned national clinical HIV registry provide the potential for the inclusion of such data for future HIV diagnoses [17]. The Danish CD4 proxy may provide a reasonable representation of the historical CD4 distribution in Norway, given the similarities in the HIV epidemics, HIV prevention and control measures, target groups for testing, the accessibility of testing, and the availability and accessibility of treatment in both countries [19, 20, 25, 27–30]. We were unable to adjust for all differences within key subpopulations in Norway and Denmark due to the small number of diagnoses in some groups (for example, see Additional file 1, Table C), thus it will be important to further validate the suitability of the proxy with future surveillance data on CD4 count from Norway and Denmark.

The main strengths of the study are that the input surveillance data were good quality national data with complete reporting, while different commonly used estimation methods have been used, at a time when the use of these tools is rapidly increasing as countries seek to monitor their own progress towards the 90–90-90 target. Also, estimates can easily be repeated to monitor progress in reducing infections and the undiagnosed populations. This study also has some limitations, including the data used for model fitting and the models themselves. It is assumed that reported data are correct, including timing of infection with regards to migration. However, a study in Sweden found that a higher proportion of migrants may be infected after migration than reported [46]. Also, clustered transmission may contrast with self-reported route of transmission, particularly among migrants from SSA [47]. There is the potential for duplicate reporting during the study period as HIV reporting was anonymised, but the risk is considered low by national experts, who have rigorously screened HIV notifications and followed up to resolve potential duplicate notifications. In the ECDC model, underreporting of AIDS diagnoses could underestimate time from infection to diagnosis. Conversely, the time from infection to diagnosis may also be fractionally overestimated in groups who test regularly, such as MSM, as the ECDC modelling tool does not account for diagnosis during the first 3 months of infection, which is feasible with current screening tests. Also, the ECDC modelling tool relies on observed data on all-cause mortality and out-migration among those diagnosed. This was unknown for 86% of input HIV diagnoses, thus PLHIV estimates are likely slightly overestimated. Collaboration with other data sources, such as a planned national clinical HIV registry, will help to validate PLHIV estimates, particularly with regards to missing outcome data. Estimates of incidence, time to diagnosis and the number undiagnosed are less affected by missing outcome data, thus one approach could be to model the number of undiagnosed infections using the ECDC modelling tool, and add this to clinical registry data [45]. In Spectrum, key limitations are the absence of CD4 data to inform the CSAVR model fitting and precise data for the number on treatment over time. While the implied mean CD4 at diagnosis over time – based on the fit to new diagnoses and AIDS deaths – appears plausible, actual data should be incorporated in the future when available. Program data were not available for the number of PLHIV on ART and estimates were instead used. Accurate numbers of those on ART can further improve the Spectrum estimates.

## Conclusion

This study has made the best use of available data, and different estimation methods, to model the HIV epidemic in Norway, in the context of the first UNAIDS 90–90-90 target. Results collectively allow cautious confidence in concluding that Norway has achieved the first UNAIDS 90–90-90 target in the general population. Results also clearly highlight the success of prevention strategies among MSM. Estimates for subpopulations strongly influenced by migration remain less clear. To further increase confidence in current estimates, future modelling should appropriately account for all-cause mortality and out-migration, either through model assumptions or in collaboration with other data sources, and adjust for time of in-migration using either precise migration data or an appropriate migration assumption for a low prevalence country.

## Supplementary information

**Additional file 1.** Further clarifications of input surveillance data.

**Additional file 2.** Model fits to data.

**Additional file 3.** Incidence estimates using the ECDC HIV modelling tool and CSAVR.

**Additional file 4.** PLHIV estimates using the ECDC HIV modelling tool, Spectrum AIDS Impact Model (with CSAVR incidence) and Spectrum AIDS Impact Model (with ECDC modelling tool incidence).

**Additional file 5.** All estimates from the ECDC HIV modelling tool, overall and by key subpopulation, using default model assumptions on CD4 distribution.

**Additional file 6.** All estimates from the ECDC HIV modelling tool, overall and by key subpopulation, including the Danish CD4 proxy.

## Data Availability

Data generated from the ECDC HIV modelling tool and Spectrum AIDS Impact Model that support the conclusions of this article are included within the article and its additional files. The input surveillance data used to run the models that support the findings of this study are available from the NIPH and Statens Serum Institute, but restrictions apply to the availability of these data for research. In order to get access to the data, permission from the NIPH or Statens Serum Institute is needed. Questions regarding access to data may be directed towards datatilgang@fhi.no (Norway) and serum@ssi.dk (Denmark). Surveillance data may also be requested from the ECDC at https://www.ecdc.europa.eu/en/publications-data/request-tessy-data-research.

## Abbreviations

ART: Antiretroviral treatment
CI: Confidence interval
CSAVR: Case Surveillance and Vital Registration
ECDC: European Centre for Disease Prevention and Control
MSM: Men who have sex with men
NIPH: Norwegian Institute of Public Health
PLHIV: People living with HIV
PrEP: Pre-exposure prophylaxis
PWID: People who inject drugs
SSA: Sub-Saharan Africa
UNAIDS: Joint United Nations Programme on HIV/AIDS
