# Supplementary material for: Monitoring progress towards the first UNAIDS 90-90-90 target in key populations living with HIV in Norway
Source: BMC Infect Dis. 2020 Jun 26;20:451. doi: 10.1186/s12879-020-05178-1 (PMC7318482; doi:10.1186/s12879-020-05178-1)
Supplement: Supplementary file 1 — Additional file 1. Further clarifications of input surveillance data. [file 12879_2020_5178_MOESM1_ESM.docx]

**Monitoring progress towards the first UNAIDS 90-90-90 target in key populations living with HIV in Norway**

**Additional file 1 – further clarifications of input surveillance data**

*Table A. Distribution of the reported route of transmission and region of birth of persons diagnosed with HIV in Norway, 1987 – 2018*

|  | **Route of transmission** | | | | | | | | | | |
| --- | --- | --- | --- | --- | --- | --- | --- | --- | --- | --- | --- |
| **Region of birth** | **Heterosexual**  **female** | | **Heterosexual**  **male** | | **MSM** | | **PWID** | | **Other/**  **Unknown**** | |  |
|  | **No.** | **%** | **No.** | **%** | **No.** | **%** | **No.** | **%** | **No.** | **%** | **Total** |
| *Europe* |  |  |  |  |  |  |  |  |  |  |  |
| Norway | 234 | 15 % | 571 | 39 % | 1314 | 73 % | 342 | 83 % | 69 | 58 % | 2530 |
| Western Europe* | 13 | 1 % | 46 | 3 % | 122 | 7 % | 17 | 4 % | 3 | 3 % | 201 |
| Central Europe | 16 | 1 % | 13 | 1 % | 48 | 3 % | 6 | 1 % | 3 | 3 % | 86 |
| Eastern Europe | 35 | 2 % | 27 | 2 % | 22 | 1 % | 27 | 7 % | 15 | 13 % | 126 |
| *Asia* |  |  |  |  |  |  |  |  |  |  |  |
| South East Asia | 272 | 18 % | 39 | 3 % | 103 | 6 % | 11 | 3 % | 11 | 9 % | 436 |
| Indian Subcontinent | 2 | 0 % | 14 | 1 % | 18 | 1 % | 0 | 0 % | 5 | 4 % | 39 |
| *Other regions* |  |  |  |  |  |  |  |  |  |  |  |
| Sub-Saharan Africa | 922 | 60 % | 704 | 49 % | 25 | 1 % | 2 | 0 % | 7 | 6 % | 1660 |
| Middle East and North Africa | 10 | 1 % | 23 | 2 % | 25 | 1 % | 7 | 2 % | 2 | 2 % | 67 |
| North America | 2 | 0 % | 2 | 0 % | 18 | 1 % | 0 | 0 % | 1 | 1 % | 23 |
| South and Central America | 33 | 2 % | 8 | 1 % | 102 | 6 % | 2 | 0 % | 3 | 3 % | 148 |
| Oceania | 1 | 0 % | 0 | 0 % | 0 | 0 % | 0 | 0 % | 0 | 0 % | 1 |
| Unknown | 0 | 0 % | 0 | 0 % | 0 | 0 % | 0 | 0 % | 1 | 1 % | 1 |
| *Total* | *1540* | *100 %* | *1447* | *100 %* | *1797* | *100 %* | *414* | *100 %* | *120* | *100 %* | *5318* |

* Western Europe excluding Norway. ** Reported route of transmission: blood (n=21), nosocomial (n=1), needle injury (n=1), other (n=2), unknown (n=95). MSM = men who have sex with men. PWID = people who inject drugs.

*Table B. Number of HIV diagnoses among persons who were previously diagnosed in another country before arrival in Norway, and thus excluded from the modelling, and who were not reported to have died or out-migrated by the end of 2018, by key subpopulation*

| **Key subpopulation** | **Number of HIV diagnoses who were previously diagnosed in another country** |
| --- | --- |
| Norwegian-born MSM | 37 |
| Migrant MSM | 118 |
| Norwegian-born heterosexuals | 44 |
| Migrant SSA-born heterosexuals | 206 |
| Migrant non-SSA-born heterosexuals | 109 |
| PWID | 35 |
| Other or unknown transmission route | 17 |
| *Overall* | *566* |

MSM = men who have sex with men. PWID = people who inject drugs.

*Table C. Distribution of the region of birth of persons diagnosed with HIV in Norway and Denmark, subpopulation migrant non-SSA-born heterosexuals, 2004 – 2018*

|  | **Reporting country** | | | |
| --- | --- | --- | --- | --- |
| **Region of Birth** | **Norway** | | **Denmark** | |
|  | **No.** | **%** | **No.** | **%** |
| *Europe* |  |  |  |  |
| Western Europe | 25 | 7 % | 42 | 18 % |
| Central Europe | 15 | 4 % | 23 | 10 % |
| Eastern Europe | 49 | 14 % | 36 | 15 % |
| *Asia* |  |  |  |  |
| South East Asia | 189 | 56 % | 66 | 28 % |
| Indian Subcontinent | 8 | 2 % | 13 | 6 % |
| *Other regions* |  |  |  |  |
| Middle East North Africa | 26 | 8 % | 35 | 15 % |
| North America | 1 | 0 % | 1 | 0 % |
| South and Central America | 24 | 7 % | 8 | 3 % |
| Oceania | 1 | 0 % | 0 | 0 % |
| Unknown | 0 | 0 % | 9 | 4 % |
| *Total* | *338* | *100 %* | *233* | *100 %* |
