# Supplementary material for: Monitoring progress towards the first UNAIDS 90-90-90 target in key populations living with HIV in Norway
Source: BMC Infect Dis. 2020 Jun 26;20:451. doi: 10.1186/s12879-020-05178-1 (PMC7318482; doi:10.1186/s12879-020-05178-1)

**Monitoring progress towards the first UNAIDS 90-90-90 target in key populations living with HIV in Norway**

**Additional file 2 – model fits to data**

**ECDC HIV modelling tool parameters and model fits to data**

*Table A. Parameters used for running the ECDC HIV modelling tool on HIV surveillance data from Norway*

| **Parameter** | **Value** |
| --- | --- |
| Number of knots | 4 |
| Diagnosis probability matrix*:  Time interval 1  Time interval 2  Time interval 3  Time interval 4 | 1980 – 1984: No serological testing  1984 – 1987: Serological testing  1987 – 1996: Significant increase in HIV testing  1996 – 2018: Combination antiretroviral treatment became available |
| Regression model | Poisson |
| Extra diagnosis rate due to symptoms associated with a CD4 count <200 at diagnosis | 0.4 |
| Adjust trend for sudden changes in most recent years | Yes |
| Bootstrap iterations | 100 |

* The diagnosis probability matrix was started on a new baseline for time intervals 2 and 3. For time interval 4, we assumed a change during the time interval, and also selected different diagnosis probabilities by CD4 categories in the models for which the Danish CD4 proxy was used. For time interval 1, no additional parameters were specified.

*Table B. Goodness-of-fit statistics for each input data item in the ECDC HIV modelling tool, including and excluding the Danish CD4 proxy, Norway*

|  | **Using default model assumptions for CD4 distribution** | | **Including Danish CD4 proxy** | |
| --- | --- | --- | --- | --- |
| **Input data item** | **Observations** | **Deviance** | **Observations** | **Deviance** |
| HIV diagnoses, total | 32 | 54.97 | 17 | 40.27 |
| HIV diagnoses, CD4 ≥500 | 0 | 0 | 15 | 14.10 |
| HIV diagnoses, CD4 350-499 | 0 | 0 | 15 | 16.79 |
| HIV diagnoses, CD4 200-349 | 0 | 0 | 15 | 18.70 |
| HIV diagnoses, CD4 <200 | 0 | 0 | 15 | 39.28 |
| HIV/AIDS diagnoses | 32 | 57.05 | 32 | 62.68 |
| AIDS diagnoses, total | 13 | 16.72 | 13 | 16.76 |
| *Total* | *77* | *128.74* | *122* | *208.59* |

*Table C. Overall goodness-of-fit statistics for ECDC HIV modelling tool in different key subpopulations, including and excluding the Danish CD4 proxy, Norway*

|  | **Using default model assumptions for CD4 distribution** | | **Including Danish CD4 proxy** | |
| --- | --- | --- | --- | --- |
| **Subpopulations** | **Observations** | **Deviance** | **Observations** | **Deviance** |
| Norwegian-born MSM | 77 | 102.73 | 122 | 126.49 |
| Migrant MSM | 77 | 96.24 | 122 | 148.54 |
| Norwegian-born heterosexuals | 77 | 65.56 | 122 | 124.67 |
| Migrant SSA-born heterosexuals | 77 | 129.29 | 122 | 310.99 |
| Migrant non-SSA-born heterosexuals | 77 | 107.14 | 122 | 178.80 |
| PWID | 77 | 76.99 | 122 | 138.95 |

**UNAIDS Spectrum AIDS Impact Model parameters and model fits to data**

*Model Parameters*

The default model parameters for developed countries in Spectrum AIDS Impact Model (AIM) v5.76 were used. This includes the default parameters for CD4 progression rates (the distribution of new infections by CD4 count and annual rate of CD4 progression), HIV mortality without antiretroviral treatment (ART) and HIV mortality with ART. Allocation of ART was assumed to be proportional to the mortality rate. The default sex ratios of incidence over time were adjusted to reflect the sex ratio of case report data (Figure A).

*Figure A. Assumptions used in Spectrum AIM for the ratio of female to male incidence over time*

*
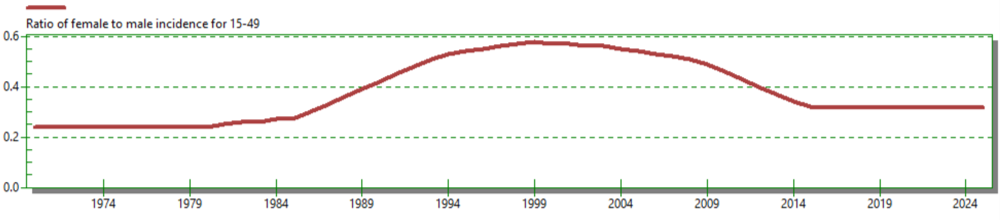
*

*CSAVR model fitting*

Models were fit to national surveillance of new diagnoses (1987-2018) and estimates of AIDS deaths adjusted for misclassification and completeness (1990-2018) produced by the Institute for Health Metrics and Evaluation for the Global Burden of Disease Study 2018 (41). Data for CD4 at diagnosis were unavailable. Mean CD4 at diagnosis is derived from the relationship inferred between the diagnosis rate and the mortality rate in the absence of treatment, and with treatment initiation, between the diagnosis rate and the treatment initiation rate.

The spline model and double logisitic model were the best fitting models based on Akaike information criterion (AIC). The model fits are pictured below in Figures B and C. The AIC values were similar for both model fits. The spline model was used for the incidence estimates as it produced a qualitatively better fit to the data in recent years.

*Figure B. CSAVR spline model fit, Norway*


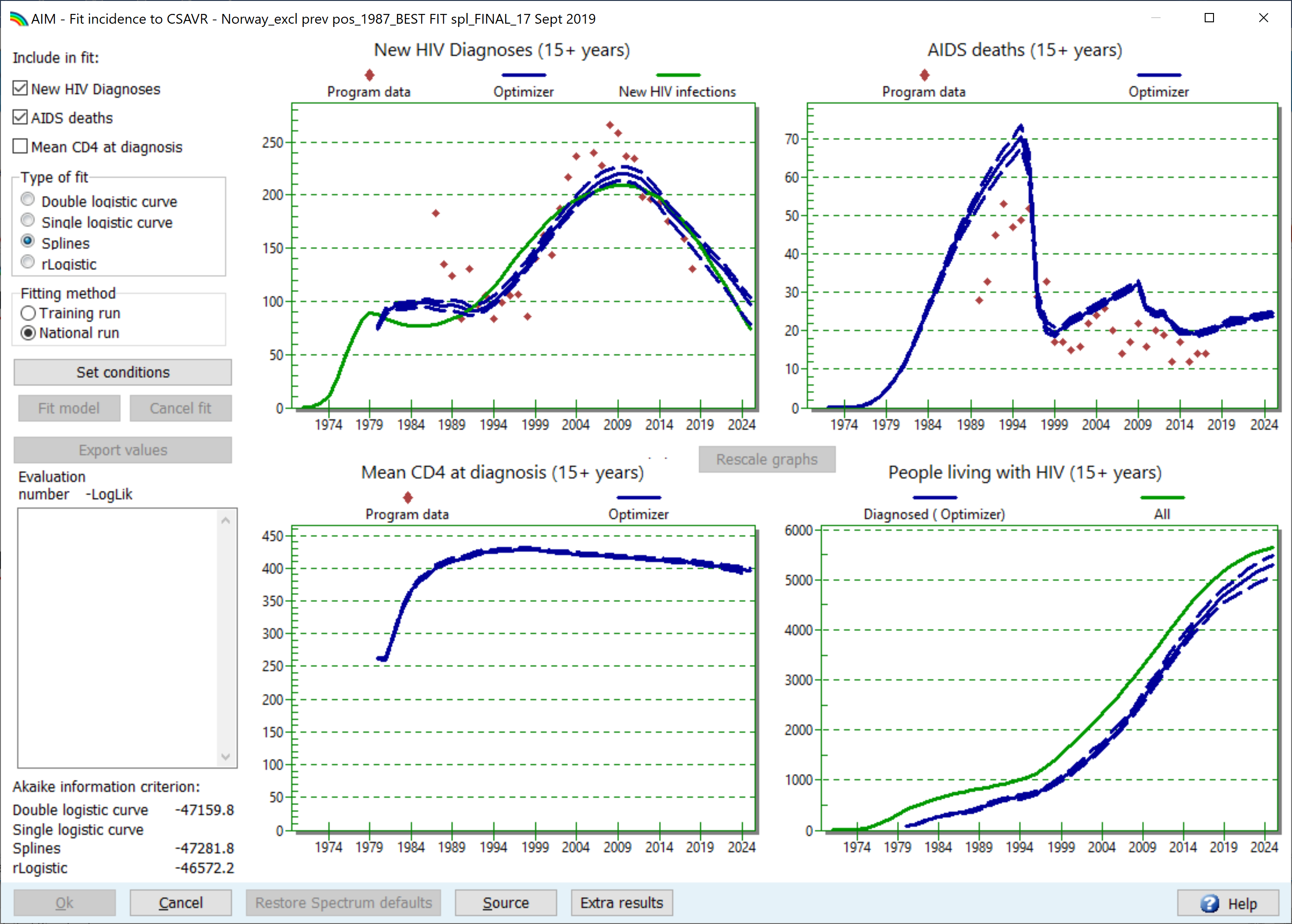


*Figure C. CSAVR double logistic model fit, Norway*


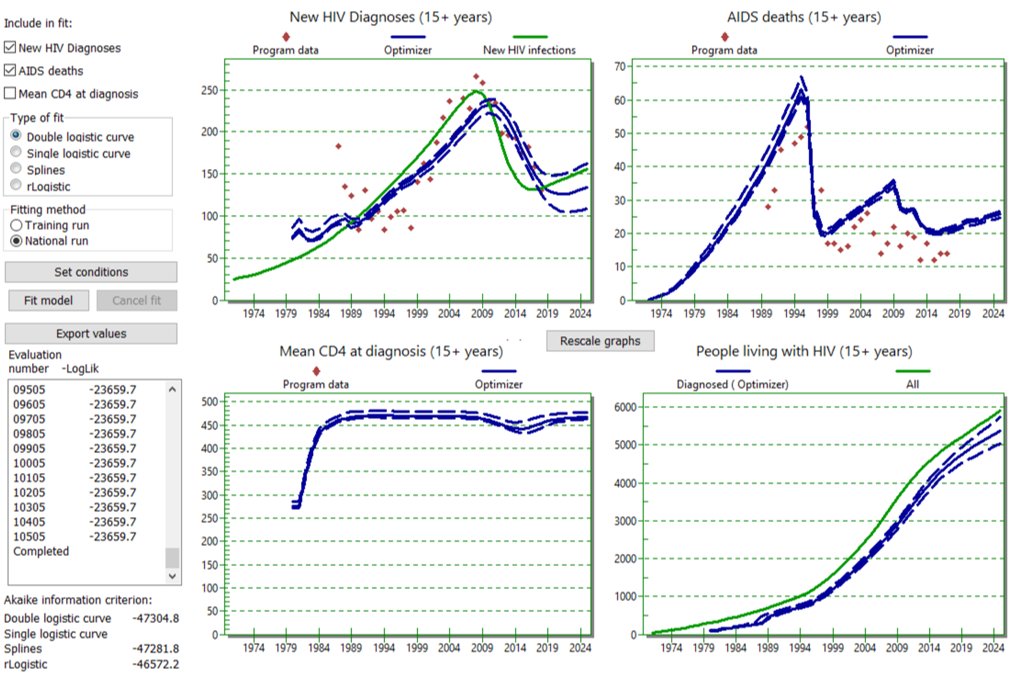

Supplement: Supplementary file 2 — Additional file 2. Model fits to data. [file 12879_2020_5178_MOESM2_ESM.docx]
